# Vasos Pequenos, Grandes Decisões

**DOI:** 10.36660/abc.20260207

**Published:** 2026-04-30

**Authors:** José Airton de Arruda, José de Ribamar Costa

**Affiliations:** 1 Hospital Evangélico de Vila Velha Vila Velha ES Brasil Hospital Evangélico de Vila Velha, Vila Velha, ES – Brasil; 2 Hospital Meridional Cariacica ES Brasil Hospital Meridional, Cariacica, ES – Brasil; 3 Hospital Unimed Vitoria Vitoria ES Brasil Hospital Unimed Vitoria, Vitoria, ES – Brasil; 4 Hospital Universitário da Universidade Federal do Maranhão São Luís MA Brasil Hospital Universitário da Universidade Federal do Maranhão, São Luís, MA – Brasil; 5 Hospital São Domingos São Luís MA Brasil Hospital São Domingos, São Luís, MA – Brasil

**Keywords:** Stents Farmacológicos, Vasos Coronários, Sirolimo, Intervenção Coronária Percutânea, Reestenose Coronária

A intervenção coronária percutânea (ICP) em vasos de pequeno calibre permanece um dos cenários mais desafiadores da cardiologia intervencionista contemporânea.

Historicamente, se considerava pequeno vaso aquele com diâmetro ≤2,75 mm. Com a evolução tecnológica dos dispositivos e o maior refinamento das técnicas intervencionistas, esse conceito tem sido progressivamente redefinido. Na prática contemporânea, foi padronizado como pequenos vasos aqueles com diâmetro <2,5 mm, enquanto os vasos muito pequenos ou extremamente pequenos incluem frequentemente segmentos <2,25 mm.^1,2^

A relevância clínica desse cenário é significativa. A doença arterial coronariana (DAC) em pequenos vasos está presente em aproximadamente 30% a 67% dos pacientes submetidos à ICP, conforme diferentes séries. Essa condição é particularmente frequente em mulheres, pacientes com diabetes mellitus e portadores de doença renal crônica, além de ocorrer com maior prevalência em segmentos distais da árvore coronária e em lesões de bifurcação, onde tanto o ramo principal distal quanto o ramo lateral frequentemente apresentam diâmetros reduzidos.^3,4^

A combinação dessas características clínicas e anatômicas cria um ambiente propício para maior risco de falha do dispositivo, restenose e necessidade de nova revascularização. Esse desafio se torna ainda mais pronunciado quando se tratam vasos extremamente pequenos. Nesses casos, o ganho luminal absoluto obtido com a intervenção é inevitavelmente limitado, e qualquer perda luminal tardia passa a ter impacto proporcionalmente maior sobre o diâmetro final do vaso.^5,6^

Assim, paradoxalmente, quanto menor o vaso, maiores podem ser as consequências das decisões terapêuticas, tornando a abordagem da DAC em pequenos vasos um cenário em que detalhes técnicos e seleção criteriosa da estratégia são determinantes para o resultado clínico.

A menor área luminal, a maior prevalência de doença difusa e as limitações técnicas inerentes aos balões e aos diferentes desenhos de stents, historicamente se associaram a taxas mais elevadas de reestenose, trombose de stent e eventos adversos clinicamente relevantes. Embora os stents farmacológicos (SF) de nova geração tenham ampliado substancialmente as possibilidades terapêuticas, esse território anatômico continua a suscitar debate, sobretudo com o ressurgimento dos balões farmacológicos ou balão revestido com medicamento (*"Drug-Coated Balloon"-* DCB) como uma alternativa pautada pelo conceito "*leave nothing behind*".^7,8^

É nesse contexto que o registro brasileiro de ICP em vasos ≤2,25 mm, comparando o stent nacional Inspiron^®^ (hastes de 75 μm e polímero biodegradável) com outros stents contemporâneos de hastes finas, oferece uma contribuição particularmente relevante.^9^ Trata-se de uma coorte de mundo real, caracterizada por alta prevalência de diabetes (42%) e síndromes coronarianas agudas (74%), baixo uso de imagem intravascular e ampla heterogeneidade de dispositivos — características que refletem fielmente a prática cotidiana, sobretudo no sistema público, onde a maioria das intervenções coronarianas no Brasil é realizada. Ademais, por ser o único estudo nacional com esse foco e metodologia, seu valor transcende o âmbito científico, assumindo importância estratégica para a tomada de decisão em cenários de recursos limitados.

**Figura 1:**
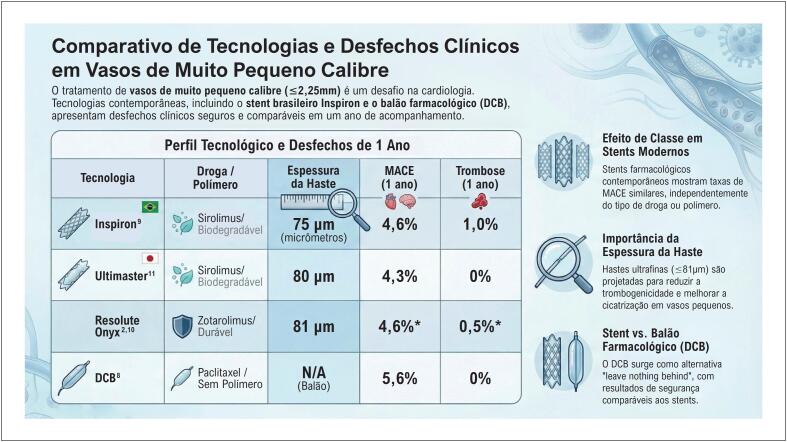
Desfechos clínicos em vasos de muito pequeno calibre (≤ 2,25 mm) em diferentes plataformas de stents e DCB. MACE: eventos cardíacos adversos maiores; DCB: Drug-Coated Balloon. *Nota: Dados do Resolute Onyx refletem a média do grupo de stents contemporâneos comparados no registro brasileiro, onde esta plataforma foi predominante.

Os resultados do registro demonstraram uma taxa de eventos cardíacos adversos maiores (MACE) de 4,6% aos 12 meses, com desempenho semelhante entre o stent Inspiron^®^ e outras plataformas contemporâneas (Figura 1). Esses achados estão em consonância com evidências internacionais sobre o uso de SF em vasos de muito pequeno calibre, incluindo séries com dispositivos eluidores de zotarolimus^2,10^ e sirolimus,^11^ que também reportaram taxas aceitáveis de eventos em médio e longo prazo. Em conjunto, os dados do estudo nacional reforçam a hipótese de um efeito de classe dos SF modernos, mesmo em um subgrupo anatômico particularmente desafiador.

Entretanto, qualquer discussão atual sobre pequenos vasos deve necessariamente incluir os DCB. O ensaio clínico randomizado PICCOLETO II^8^ demonstrou menor perda luminal tardia (*late lumen loss* — LLL) aos 6 meses com DCB em comparação ao stent eluidor de everolimus, resultado frequentemente interpretado como superioridade angiográfica da estratégia baseada em balão. Essa conclusão, no entanto, merece análise cautelosa. Ao comparar estratégias de balão versus stent, o LLL se mostra um desfecho primário inadequado, por não considerar as diferenças fundamentais no ganho luminal agudo entre as modalidades. Nos primeiros ensaios randomizados com stents metálicos (BENESTENT e STRESS),^12^ se observou maior LLL com stents em relação à angioplastia com balão, mas ainda assim os stents foram superiores, pois o maior ganho luminal imediato mais do que compensava a perda tardia. No PICCOLETO II,^8^ o ganho agudo foi aproximadamente 50% maior no grupo stent, tornando previsível uma maior LLL nesse braço. Assim, se pode considerar que o parâmetro clinicamente relevante é o diâmetro luminal no seguimento, e não a proporção do ganho luminal inicial que foi perdida. De fato, no próprio estudo, o diâmetro luminal mínimo e a porcentagem de estenose no seguimento não diferiram entre os grupos, o que enfraquece qualquer alegação robusta de superioridade do DCB. Além disso, apenas cerca de 3% dos pacientes apresentaram morte ou infarto em 12 meses, ressaltando que o estudo é subdimensionado para avaliar segurança clínica, e que seriam necessários ensaios muito maiores para comparar de forma confiável as duas estratégias. Assim, embora os DCB representem uma alternativa promissora e conceitualmente atraente em vasos pequenos, a evidência disponível permanece heterogênea, dependente do dispositivo e fortemente influenciada pela escolha dos desfechos, devendo ser interpretada com prudência quando comparada aos DES contemporâneos.

Essa discussão também levanta uma questão metodológica e clínica relevante: qual é, afinal, a melhor forma de avaliar o sucesso terapêutico em vasos muito pequenos? Desfechos angiográficos clássicos, como LLL ou mesmo reestenose angiográfica, podem ter significado clínico limitado nesse território. Em vasos de pequeno calibre, a quantidade absoluta de miocárdio irrigado é frequentemente menor, e a recorrência de estenose nem sempre se traduz em sintomas ou eventos clínicos relevantes. Essa dissociação entre achados angiográficos e manifestações clínicas pode, em parte, explicar por que algumas estratégias terapêuticas apresentam desempenho clínico aparentemente melhor do que o sugerido pelos parâmetros angiográficos isolados — sobretudo quando não há reestudo angiográfico sistemático.

Essa observação suscita ainda outra reflexão prática: será que devemos tratar todos os vasos pequenos? Em muitos casos, a decisão pode se beneficiar de uma seleção mais criteriosa, integrando o contexto clínico, a presença de síndrome coronariana aguda e a avaliação funcional com fisiologia intracoronária, especialmente nas situações de doença coronária crônica. Estratégias guiadas pela pesquisa de substrato isquêmico por meio de métodos invasivos podem ajudar a identificar quais lesões em vasos pequenos realmente têm relevância funcional, evitando intervenções potencialmente desnecessárias em territórios de menor impacto prognóstico.

No que diz respeito às opções terapêuticas, além dos SF e dos DCB, vale mencionar abordagens alternativas desenvolvidas especificamente para esse cenário anatômico. Entre elas, se destaca o conceito de "*Stent-on-a-wire*", representado pelo sistema Svelte^®^, projetado para simplificar o procedimento e potencialmente reduzir o perfil do dispositivo em vasos pequenos. A experiência nacional com essa tecnologia foi relatada no SISC Trial,^13^ conduzido por Chamie et al., demonstrando a viabilidade dessa estratégia em um contexto de prática real.

É justamente nesse cenário que o registro brasileiro ganha relevância adicional. Ao demonstrar que, mesmo em um ambiente de alta complexidade clínica e com uso mínimo de imagem intravascular, os SF modernos apresentam resultados clínicos consistentes em vasos muito pequenos, o estudo fornece um referencial pragmático para centros onde o acesso a DCB específicos ou a estratégias híbridas pode ser limitado. Em outras palavras, enquanto os DCB representam uma alternativa conceitualmente atraente e sustentada por ensaios randomizados selecionados, os SF contemporâneos continuam sendo uma opção previsível e segura no mundo real.

Do ponto de vista metodológico, o caráter observacional e a ausência de randomização impõem limitações conhecidas, assim como o seguimento de 12 meses é mais curto do que o disponível em algumas séries internacionais.^2,11^ Ainda assim, essas características são também parte da força do estudo: ele captura a prática como ela é, e não como seria em ambientes ideais.

Em síntese, o conjunto das evidências atuais sugere que vasos muito pequenos deixaram de ser território proibido tanto para SF quanto, em casos selecionados, para DCB. O registro brasileiro não concorre com os ensaios randomizados; ao contrário, ele os complementa, oferecendo a perspectiva do mundo real em um sistema de saúde com desafios próprios. Ao fazer isso, reforça a importância da produção científica nacional e amplia a base de evidências para decisões individualizadas no laboratório de hemodinâmica. Mais do que discutir qual dispositivo é superior, talvez o desafio contemporâneo esteja em definir melhor quais vasos pequenos realmente precisam ser tratados e quais desfechos devem orientar nossas decisões clínicas. Afinal, quando se trata de intervenção coronária em pequenos vasos, decisões aparentemente pequenas podem ter grande impacto clínico.
