# Supplementary material for: Vasos Pequenos, Grandes Decisões
Source: Arq Bras Cardiol. 2026 Apr 30;123(2):e20260207. [Article in Portuguese] doi: 10.36660/abc.20260207 (PMC13155789; doi:10.36660/abc.20260207)
Supplement: Translation

# Translation: Small Vessels, Big Decisions

**DOI:** 10.36660/abc.20260207i

**Authors:** José Airton de Arruda, José de Ribamar Costa

**Affiliations:** 1 Vila Velha ES Brazil Hospital Evangélico de Vila Velha, Vila Velha, ES – Brazil; 2 Cariacica ES Brazil Hospital Meridional, Cariacica, ES – Brazil; 3 Vitoria ES Brazil Hospital Unimed Vitoria, Vitoria, ES – Brazil; 4 São Luís MA Brazil Hospital Universitário da Universidade Federal do Maranhão, São Luís, MA – Brazil; 5 São Luís MA Brazil Hospital São Domingos, São Luís, MA – Brazil

**Keywords:** Drug-Eluting Stents, Coronary Vessels, Sirolimus, Percutaneous Coronary Intervention, Coronary Restenosis

Percutaneous coronary intervention (PCI) in small-caliber vessels remains one of the most challenging scenarios in contemporary interventional cardiology.

Historically, a small vessel was considered to be one with a diameter ≤2.75 mm. With the technological evolution of devices and the greater refinement of interventional techniques, this concept has been progressively redefined. In contemporary practice, small vessels are standardized as those with a diameter <2.5 mm, while very small or extremely small vessels often include segments <2.25 mm.^1,2^

The clinical relevance of this scenario is significant. Small vessel coronary artery disease (CAD) is present in approximately 30% to 67% of patients undergoing PCI, according to different series. This condition is particularly frequent in women, patients with diabetes mellitus, and those with chronic kidney disease, and occurs more commonly in distal segments of the coronary tree and in bifurcation lesions, where both the distal main branch and the side branch often have reduced diameters.^3,4^

The combination of these clinical and anatomical characteristics creates an environment conducive to a higher risk of device failure, restenosis, and the need for repeat revascularization. This challenge becomes even more pronounced when dealing with extremely small vessels. In these cases, the absolute luminal gain obtained with the intervention is inevitably limited, and any late luminal loss has a proportionally greater impact on the final vessel diameter.^5,6^

Thus, paradoxically, the smaller the vessel, the greater the potential consequences of therapeutic decisions, making the approach to CAD in small vessels a scenario where technical details and careful strategy selection are crucial for the clinical outcome.

The smaller luminal area, the higher prevalence of diffuse disease, and the inherent technical limitations of balloons and different stent designs have historically been associated with higher rates of restenosis, stent thrombosis, and clinically relevant adverse events. Although new-generation drug-eluting stents (DES) have substantially expanded therapeutic possibilities, this anatomical territory continues to generate debate, especially with the resurgence of drug-coated balloons (DCBs) as an alternative based on the "leave nothing behind" concept.^7,8^

It is in this context that the Brazilian PCI registry in vessels ≤2.25 mm, comparing the national Inspiron^®^ stent (75 μm struts and biodegradable polymer) with other contemporary thin-strut stents, offers a particularly relevant contribution (ABC-2025-0145).^9^ This is a real-world cohort, characterized by a high prevalence of diabetes (42%) and acute coronary syndromes (74%), low use of intravascular imaging, and wide heterogeneity of devices—characteristics that faithfully reflect daily practice, especially in the public system, where most coronary interventions in Brazil are performed. Furthermore, being the only national study with this focus and methodology, its value transcends the scientific realm, assuming strategic importance for decision-making in resource-limited settings.

The registry results demonstrated a MACE rate of 4.6% in 12 months, with similar performance between the Inspiron^®^ stent and other contemporary platforms (Figure 1). These findings are consistent with international evidence on the use of DES in very small-caliber vessels, including series with zotarolimus^2,10^ and sirolimus^11^ eluting devices, which also reported acceptable rates of medium- and long-term events. Taken together, the data from the national study reinforce the hypothesis of a class effect of modern DES, even in a particularly challenging anatomical subgroup.

**Figure 1:** Clinical outcomes in very small caliber vessels (≤ 2.25 mm) on different stent platforms and DCB. MACE: Major adverse cardiovascular events; DCB: Drug-Coated Balloon. *Note: Data from Resolute Onyx reflect the average of the group of contemporary stents compared in the Brazilian registry, where this platform was predominant.

However, any current discussion about small vessels must necessarily include DCBs. The randomized clinical trial PICCOLETO II^8^ demonstrated less late lumen loss (LLL) at 6 months with DCB compared to everolimus-eluting stents, a result often interpreted as angiographic superiority of the balloon-based strategy. This conclusion, however, deserves cautious analysis. When comparing balloon versus stent strategies, LLL proves to be an inadequate primary endpoint, as it does not consider the fundamental differences in acute luminal gain between the modalities. In the first randomized trials with metallic stents (BENESTENT and STRESS),^12^ a higher LLL was observed with stents compared to balloon angioplasty, but stents were still superior, as the greater immediate luminal gain more than compensated for the late loss. In the PICCOLETO II^8^ study, the acute gain was approximately 50% greater in the stent group, making a higher LLL in that arm predictable. Thus, the clinically relevant parameter can be considered to be the luminal diameter at follow-up, and not the proportion of initial luminal gain that was lost. In fact, in the study itself, the minimum luminal diameter and the percentage of stenosis at follow-up did not differ between the groups, which weakens any robust claim of DCB superiority. Furthermore, only about 3% of patients experienced death or myocardial infarction at 12 months, highlighting that the study is undersized to assess clinical safety and that much larger trials would be needed to reliably compare the two strategies. Thus, although DCBs represent a promising and conceptually attractive alternative in small vessels, the available evidence remains heterogeneous, device-dependent, and strongly influenced by the choice of outcomes, and should be interpreted with caution when compared to contemporary DES.

This discussion also raises a relevant methodological and clinical question: what is, after all, the best way to assess therapeutic success in very small vessels? Classic angiographic outcomes, such as LLL or even angiographic restenosis, may have limited clinical significance in this territory. In small-caliber vessels, the absolute amount of irrigated myocardium is often smaller, and recurrence of stenosis does not always translate into relevant symptoms or clinical events. This dissociation between angiographic findings and clinical manifestations may, in part, explain why some therapeutic strategies show apparently better clinical performance than suggested by isolated angiographic parameters — especially when there is no systematic angiographic restudy.

This observation raises another practical question: should we treat all small vessels? In many cases, the decision can benefit from a more careful selection, integrating the clinical context, the presence of acute coronary syndrome, and functional assessment with intracoronary physiology, especially in situations of chronic coronary artery disease. Strategies guided by the search for ischemic substrate through invasive methods can help identify which lesions in small vessels are truly functionally relevant, avoiding potentially unnecessary interventions in areas with less prognostic impact.

Regarding therapeutic options, in addition to DES and DCB, it is worth mentioning alternative approaches developed specifically for this anatomical scenario. Among them, the "Stent-on-a-wire" concept stands out, represented by the Svelte^®^ system, designed to simplify the procedure and potentially reduce the device profile in small vessels. National experience with this technology was reported in the SISC Trial,^13^ conducted by Chamie et al., demonstrating the feasibility of this strategy in a real-world practice context.

It is precisely in this scenario that the Brazilian registry gains additional relevance. By demonstrating that, even in a highly complex clinical environment and with minimal use of intravascular imaging, modern DESs present consistent clinical results in very small vessels, the study provides a pragmatic benchmark for centers where access to specific DCBs or hybrid strategies may be limited. In other words, while DCBs represent a conceptually attractive alternative supported by selected randomized trials, contemporary DESs remain a predictable and safe option in the real world.

From a methodological point of view, the observational nature and the absence of randomization impose known limitations, as does the 12-month follow-up, which is shorter than that available in some international series.^2,11^ Still, these characteristics are also part of the study's strength: it captures practice as it is, not as it would be in ideal settings.

In summary, the current body of evidence suggests that very small vessels are no longer off-limits territory for both DES and, in selected cases, DCB. The Brazilian registry does not compete with randomized trials; on the contrary, it complements them, offering a real-world perspective within a healthcare system with its own challenges. In doing so, it reinforces the importance of national scientific production and expands the evidence base for individualized decisions in the hemodynamics laboratory. More than discussing which device is superior, perhaps the contemporary challenge lies in better defining which small vessels truly need to be treated and which outcomes should guide our clinical decisions. After all, when it comes to coronary intervention in small vessels, seemingly small decisions can have a large clinical impact.
